# Critical elements in implementations of just-in-time management: empirical study of cement industry in Pakistan

**DOI:** 10.1186/2193-1801-2-645

**Published:** 2013-12-01

**Authors:** Muhammad Imran Qureshi, Mehwish Iftikhar, Mansoor Nazir Bhatti, Tauqeer Shams, Khalid Zaman

**Affiliations:** Department of Management Sciences, COMSATS Institute of Information Technology, Abbottabad, Pakistan

**Keywords:** Just-in-time management, Production process, Structural equation model, Cement industry, Pakistan

## Abstract

In recent years, inventory management is continuous challenge for all organizations not only due to heavy cost associated with inventory holding, but also it has a great deal to do with the organizations production process. Cement industry is a growing sector of Pakistan’s economy which is now facing problems in capacity utilization of their plants. This study attempts to identify the key strategies for successful implementation of just-in-time (JIT) management philosophy on the cement industry of Pakistan. The study uses survey responses from four hundred operations’ managers of cement industry in order to know about the advantages and benefits that cement industry have experienced by Just in time (JIT) adoption. The results show that implementing the quality, product design, inventory management, supply chain and production plans embodied through the JIT philosophy which infect enhances cement industry competitiveness in Pakistan. JIT implementation increases performance by lower level of inventory, reduced operations & inventory costs was reduced eliminates wastage from the processes and reduced unnecessary production which is a big challenge for the manufacturer who are trying to maintain the continuous flow processes. JIT implementation is a vital manufacturing strategy that reaches capacity utilization and minimizes the rate of defect in continuous flow processes. The study emphasize the need for top management commitment in order to incorporate the necessary changes that need to take place in cement industry so that JIT implementation can take place in an effective manner.

## Introduction

In the face of current economic crunch, companies are looking for the ways to cope with the situation by opting for cost reduction and quality products at the same time. Referring back to Japanese manufacturing success in 1980s, companies find the TQM and just-in-time (JIT) inventory management systems are some of most popular ways to have lower cost and high quality products (Daniel and Reitsperger 1996). Slack et al. (2007) defined JIT as an operations concept, which focuses on meeting the demand while offering the perfect quality and zero waste.

Advantages of implementing JIT are enormous. (Klein and Devens 1999) argued that it leads to efficiency and effectiveness. JIT increases communication inside the organization as well as outside the organization with other organizations such as vendors and distributors (Inman and Mehra 1991). JIT implementation also leads to the reduction in the cost of purchase which has been the major expense for many industries (Gargeya and Thompson 1994). Just in time tries to promote managerial involvement and organizational discipline (Ptak 1991; Bolander et al. 1999). JIT also tends to combine the different organizational functional areas. It specially endavour to make connection between accounting and production (Johansson 1990; Sandwell and Molyneux 1989; Green et al. 1991; Bhimani and Bromwich 1991). Biggart and Gargeya (2002) found that JIT implementation helps to minimize the amount of work-in-process inventory, raw material and the finished goods. Thus all these advantages are contributing in lowering the costs of production and the product itself.

However, implementation of JIT has posed many setbacks to the firms who are actually following this philosophy. For example, Japanese faced several problems while implementing this philosophy such as suppliers have been blamed for inconsistency in the delivery process due to traffic problems. Some experts also blamed that JIT philosophy switches the responsibility of this inefficiency from more powerful and large manufacturing companies to smaller, lesser powerful vendors. JIT is also vulnerable in the management of natural catastrophes such as earthquakes, floods, storms etc. as evidenced by the Great Hanshin Earthquake in Japan when deliveries were stopped to the facilities of Toyota although the factories were not damaged at all (Daniel and Reitsperger 1996).

Beyond these above mentioned barriers to the successful implementation of JIT approach, companies may also find problems due to gaps between the communication facilities available to manufactures and suppliers. Proper training of the employees as well as the top management involvement is the important factors for the successful implementation of JIT (Minahan 1996). Presence of accurate data including the accurate and reliable forecast of demand is a key for JIT to operate smoothly (Francis 1989). Given the potentials of the JIT, implementation of this philosophy will be of great help to Pakistani companies in this current economic downturn.

Pakistan cement industry has exposed marvelous development since the time of independence. In the year 1947, there were just 4 operational cement units in West Pakistan having round about half a million tons per annum of production capacity. Total demand through the same period was estimated at over a million tones. The industry showed gradual growth as in 1950’s, only 5 plants were set up with a capacity of 2.8 million tons in total with 4 more installations in the 1960’s (Lewis and Soligo 1965). The construction industry passed through a boom as demand increased because of an outgrowing economy and by 1969 the Pakistan’s cement industry had 14 operational cement plants having capacity of 3.3 million tones (annual). For the duration of these 3 decades, BY 1992 production went up from 3.5 million tons to 8.4 million tons and cement requirement of Pakistan’s cement was largely been met with exports which has been started in 1977 and remained continue until 1995. And in 1977 to 1988 Government policy of Pakistan moved towards denationalization and had complete focus towards construction and housing. In 1980s in order to meet demand, the government permitted to set up 7 more units by the private sector housing units having total capacity of 2.54 million tones and four more plants were made operational by the SCCP in public sector, resulting at about 24 operational cement plants in Pakistan, by the end of this period. On the other hand, there were huge price differentials between public and private sector manufacturing units in Pakistan as SCCP i-e the scientific committee on consumer products fixed cement prices much lower for the public sector companies. During to 1995, in hand capacity of the cement plants was not able to meet the local demand mainly in the north of Pakistan resulting an imminent and enormous need of increasing the in hand capacity of the cement plants in order to satisfy the growing need. At the same time a few plants were also shut down due to different reasons resulting the dramatic increase in the prices in 1990s. The shortage of local cement and high cost of import were a few main reasons behind this huge increase in the price at that time. In the world and the local economy, by the projections for accelerated growth in demands, 5 more plants were set up to satisfy cement requirements at local level. Still, the local demand didn’t expend in compatible to its growth during 1995 to 2000. The cement sector experienced growth rates of 8% per annum which was very low. Consequently in post-industry expansion of 90’s, manufacturers of cement had to go through a challenging period of capacity utilization, Pakistan began exporting in the years 2001 to 2002 for utilizing excess capacity. In the recent years, capacity utilization of the cement industry is only 64% approximately. To overcome this problem, cement industry should rethink on the reduced deficits and focus on infrastructure building to meet the market demand and to maximize profit from its operations. Just-in-time (JIT) management philosophy focuses on the reduction of wastes and improves the efficiency of the manufacturing process. Same problem exists in the cement industry of Pakistan and the manufacturers are continuously looking the ways to reduce defects and efficient inventory management to increase the capacity utilization during the production processes.

### Research objectives

The objective of the study is to identify the key strategies for successful implementation of JIT i-e just-in-time management philosophy on cement industry of Pakistan by using survey responses from four hundred operations’ managers of cement industry. The most imminent barriers to the successful implementation of JIT in Pakistan are electricity crisis, terrorism, natural catastrophes, economic crises, technology gap between the power manufacturers and the weak suppliers to name a few. Keeping in view, the more specific objectives as follows:

I. To investigate the imminent factors which somehow influences the cost associated with production process, reduce the inventory costs and smooth running of production process in cement industry of Pakistan.
II. To identify those factors which eliminate and reduce the waste of resources; inappropriate processes and redundant waiting time in the production processes.

The main building blocks of the study are divided in the following sections. After introduction which build up Section 1, Section two discusses the review of literature. Methodological and results are discussed in Section 3. Final section concludes the study.

## Literature review

The manufacturers operating in the moeren era face a lot of challenges among them the factors of most pivotal focus are making operations faster, customer service improvement, and cost reduction. In order to compete globally, US companies are looking for new ways for improving their abilities. during the past decade a technique that has grabbed a greater attention in order to compete globally is the concept of Just-in-time (Zhu et al. 1994). Wafa and Yasin (1998) pointed out that JIT is a continuous goal oriented process in order to remove waste and increase productivity. They also mentioned that JIT is used for the description of manufacturing system where different parts are produced that are essential to complete finished products or delivered where needed. In the past, “JIT was considered to be an inventory reduction mechanism that can be used to decrease the levels of inventory in a production process continually until it is stopped by some occurrence (Spencer et al. 1994). Missing to which the firm may encounter some of the problems like losing market shares, high scrap, high levels of inventory, low quality in labor and products, longer lead times and the survival of many sources of waste in the process of production (Salaheldin and Francis 1998). Studies proved that the successful implementation of JIT can help better to lessen many of the obstacles that are mentioned above (Vuppalapati et al. 1995; Draper 1995; Walley 2000; Cua et al. 2001). Though, relevant literature review reveals that the philosophy of successful implementation of JIT is based mainly on the efforts regardingseveral modifications that are to be undertaken before this implementation process. One major change that should be undertaken before the JIT implementation is that it demands a major change in the attitudes of the people and work habits as well (Gupta 1990; Norris and Swanson 1994; Yasin et al. 2001).

Some literature has shown the positive impacts of JIT when applied on the strategic and operational aspects of an organization especially in the private sector. Yasin et al. (2001) conclude that JIT organizational strategic philosophy can increase the effectiveness and efficiency of organizations (Vokurka and Davis 1996; Klein and Devens 1999). The phenomina explaining the importance of JIT and its efficacy for the modern organizations can easily be understoor from the concluding remarks of Pandya and Boyd (1995) i.e., the most successful Japanese companies in financial terms that are working in the UK are those which have cellular type manufacturing, operate total quality control and have a JIT approach. Shin and Min (1991) and Yasin and Wafa (1996) concludes that JIT has significant and positive impacts on US businesses. JIT improves communication between and within an organization, and its vendor and customers (Inman and Mehra 1991). It may also remove the waste in production process (Tesfay 1990).

Practitioners and researchers indentified that several modifications in the existing systems should be undertaken before the implementation of JIT. More important is that the JIT needed a modified approach from top management which involve significant modifications i-e designing such type of organization that integrates strategy with people for achieving the basic premise of JIT, waste elimination, reducing organization and specialization functions, making everyone responsible for production of quality services and products, developing project teams, promoting management and employees’ commitment for continuous improvement (Theng 1993; and Chong et al. 2001); also combining organizational and HR systems with hardware (Sim 2001). Secondly, for the engineering of JIT modifications the organization need to incorporate some important changes which may involve the need forcombining several operations for minimizing the distance traveled; changing work center layout; combining machines in cells; buying equipment with short setup (Wafa and Yasin 1998); product design responsibility; reliability and quality; using experiment designs for improving quality inorder to succeed in the cost reduction. The adaptation of the machanisim also encourages the manufacturing unit for looking for product standardization whereever feasible; to concentrate on continuous progress in product design (Theng 1993); using TPM (total productive maintenance) as an integral part of a JIT system (Bamber et al. 2000). Furthermore the implementation requires more sophisticated operations where the implemented operational machanisims are in anycase re-analyzed for the successful implementation for identifying the needed adjustments where simplifications, standardization and automation are required (Yasin et al. 2001). Worth mentioning that the success of JIT also depends on design of the product in any business. Tan (2001) shows that JIT strategy influences product design and development strategies significantly. On the basis of above cited literature, this study posits that product design affect JIT implementation positively:

It is very important, to mention about the difficulties during the implementation of JIT since the conception stage of the implementation the top management should wholeheartedly be into it and in an agreement for this implementation recognizing it as the most important strategic consideration and intent to reduce the cost and increase the overall profitability of the firm. Monitoring the whole process is another important aspect as it is pivotally needed to observe how efficiently and smoothly the process is being implaced. Kazazi (1994) and Banerjee and Kim (1995) opened that cooperation among buyer and vendor is an imperative ingredient for effective and successful implementation of JIT. Zhu et al. (1994) also concluded that human related practices are also important like relationship among coworkers and communication between workers, and the interpersonal skills for the effective implementation of JIT. So employee commitment during the implementation process is needed most, which can be increased by educating the employees first about the overall process of JIT and their responsibilities i-e by making them aware about their contributions to JIT implementation process. On the other way around, Inman and Mehra (1991) survey findings do not show the significant relationship between JIT educational strategy and management commitment and successful implementation of JIT.

JIT requires significant modifications during its implementation it is like designing a model organization which could transform itself completely by integrating strategy with people to achieve JIT objectives, i.e. eliminating waste, making teams, and making each worker responsible for the production of quality products as well as quality services in order to meet total quality control criteria (Theng 1993; and Chong et al. 2001).

Inman and Mehra (1991) determined that TQM is an important aspect of JIT implementation. TQM principle is implemented on each and every worker in an organization who should be involved throughout the process particularly related to the improvement of products and services in terms of quality but as mentioned earliar this process is mainly dependent upon the top management commitment. TQM (Total quality management) has widened the production process to the whole company and suppliers not only manufacturing. In Japan, quality controls is an amelgimation of some diverse activities ranging from research and development, top management support, purchasing, finance, marketing, and all the aspects of facility operations. In the final stages of implementation after the completion of the necessary training, formation of team and thegoal setting the implementation of the JIT or TQM system is good to go. Total quality management in the US has the similar aspacts but they are implemented differently than implementation in Japanese firms.

Reducing defects or increasing the quality of products is an important aspect of JIT initiative. In the context of JIT, quality has vital importance as conformance to standard because it is the quality control that contributes in minimizing the variance of products distributed around the mean Reeves & Bednar (1994). Thus we advance the following hypothesis:

Spencer and Guide (1995) conducted a survey and asked several questions regarding the inventory management aspects of JIT from the respondents divided into two groups. Results showed that both the groups were not agreed with the idea that JIT is largely a matter of inventory reduction but agreed on that its goal is the removal of non value added activities. This study also suggests that Quality is one of the important components of JIT implementation. This study identified some of the important elements that are essential for the successful implementation of JIT mechanisms like proper physical resource management that include reduction of setup and preventive maintenance, human resource and quality management. The understanding of the importance of inventory management by all the levels of organization is essential for the core philosophy of JIT. Salaheldin (2005) study also supports the above argument that for the successful implementation of JIT philosophy, effective modifications are necessary for inventory management as well as for purchasing methods. For this purpose the openness of communication between management and employees is the pivotal necessity.

The implementation of JIT can assert marvelous impacts on different factors like production lead time, cost of labor, inventory level and manufacturing space requirement, only when it is implemented correctly. Its effectiveness mostly depends on the technique used while implementation (Groebner and Merz 1994). JIT management theory is a wide concept of business and is related to inventories directly. But it is not the whole story yet. Just in time process is the removal of waste including dead inventory, but also including scrap, indirect labor, rework, activities that are not value adding for the firm, machines that are non-productive and the quality of materials as well. The impact on labor and cost controls are also evidently seen. Inventory reduction is only the reduction of cost of in hand inventory to a satisfactory levels, with a least amount of safety level for definite unexpected cycles or demands. There are lot of different methods used for inventories forecasting like product order quantity, quantity of economic order, and discount models of quantity that may trim down the amount of cost that is included in the inventories themselves contributing towards the savings of capital. We can draw another hypothesis on the basis of above discussion i.e.

The proposal of implementing Just in time (JIT) practices upstream with the supply chain is possibly as old as the concept of JIT itself. Regarding the impact of JIT supply practices, many authors have the same opinion that implementation of JIT at the manufacturer supplier interface may contribute to the production planning processes, which significantly streamline procurement processs and this efficiency results in cost saving and smoothening the material flow (Jones et al. 1997; Lamming 1993; Helper 1991). Mistry (2005) have conducted some interviews in an electronics manufacturing company and identified that, in addition to inventory reduction, a further benefit of the important through the supplier delivery program of JIT was obtaining activities simplification for the manufacturers of the firm. After implementation of JIT supply material handlers requirement at the buying company’s plant were no longer, with the personnel salaries resulting savings.

The findings of Mackelprang and Nair (2010) reveled that JIT deliveries are positively associated with inventory from the suppliers, delivery performance and cycle time. Numerous studies have been conducted so far regarding JIT techniques in the context of Pakistan. Irfan et al. (2008) identified the problems of Pakistani firms regarding supply chain management in order to improve their overall performance and competitive positions. They found that regional level suppliers need to interact with manufacturers using JIT approach in order to enhance the effectiveness of supply chain. From the above discussion, the study posit the following hypothesis i.e.,

While probing into the current scenario of automotive manufacturing industry its productivity and possible remedies to improving the productivity, Sarwar et al. (2012) found that the effective usage of technology can enhance productivity. Tanveer et al. (2012) during the investigation of declining market share of Pakistani football manufacturing industry mentioned that customers prefer those suppliers who can adopt quickly to new technology and simplified processes for production, cost reduction and those who possess the JIT capabilities with lower inventory. JIT usually reduce the lead-time, improve quality of production, increases productivity and increases customer responsiveness (Crawford and Cox 1991; Green et al. 1991; Arogyaswamy and Simmons 1991; Cook 1996).

Another survey study reported the benefits of JIT that include reduction in inventory, minimizing the lead time, quality improvement, and better equipment and employee utilization. Provided with the effective implementation of JIT process if not then it will be difficult to get the desirable benefits (Zhu et al. 1994).

JIT also require changes in material flow by modifying inventory, production and other policies as well as reduction in number of vendors (Wafa and Yasin 1998). Stabilizing the daily or weekly production schedules is also very important. Planning production and creating different methods for estimating work in process and to identify its need is also pivotally important (Theng 1993). One other aspact during the implementation process can not be neglected and that is reduction in lot sizes under production and timed delivery machanisims are beneficial for both buyer and the vendor. It is also an undeniable fact that the smaller the production lotlarger will be the flexibility in scheduling and the capacity utilization (Banerjee and Kim 1995).

Daniel and Reitsperger (1996) revealed that by improving manufacturing flexibility and decreasing in-process inventories JIT enhances reduction in lot sizes capabilities of a firm. It is essential during implementation to continuously monitor the production plans in order to recognize the importance of JIT mechanisms.. JIT production process refers to the adoption of practices aiming at the reorganization of shop floor and streamlining production flows within production plants (Furlan et al. 2010), JIT production (Inman and Mehra 1991) and Internal JIT (Furlan et al. 2010). Some of the commonly used JIT production practices involve set up time reduction, daily schedule adherence, small lot size, kanban based pull systems, cell layout (u-shapped) and heijunka boxes (Mackelprang and Nair 2010; Motwani 2003). JIT supply is a practical implementation of the pul logic (Sakakibara et al. 1997) where efficiency is obtained through suppliers by ensuring frequent and unintrupted delivery and that too in small lots. Most of the authors argued that JIT production needed low inventories and fast throughputs and JIT supply is critical for the maintenance of the continuous flow of raw materials or components from upstream (Hsu et al. 2009; Panizzolo 1998). Even though this confirmation is suggesting that JIT supply and production are severely interrelated practices and that operations can be benefited from joint implementation of them, empirical studies are lacking in investigating this linkage. As with respect to the effect of JIT production practices, research shows that set up time minimization, concurrent use of cellular manufacturing, pull systems and adherence of daily schedule provides a continuous material flow that is to be achieved throughout the production lines, hance reducing work in-process inventory and unnecessary delays in flow time. It further contributes in minimizing the manufacturing costs, speeding up the core activities and ensuring on-time delivery performance (Brown and Mitchell 1991; Manoocheri 1984). Mackelprang and Nair (2010) reveal that the positive association between JIT production, delivery performances and efficiency is generally emphasized in various empirical studies. So, we can hypothesize that the implementation of JIT production practices may have a positive impact on operational performance.

Gyampah and Gargeya (2001) have contributed to the litriture by conducting a study on the process of implementation of just-in-time (JIT) in manufacturing firms of Ghana. They came to know that there is huge difference between JIT firms and non-JIT firms in terms of their efforts for set up time reduction, suppliers’ partnership and the training of employees for ensuring continuous quality improvements.. Though, there is no significant difference pertaining to the use of measurement systems.

On the basis of above discussions on the said topic, the current study focus on how to reduce manufacturing costs for the cement industry in Pakistan and attempt to find the factors which are important to reduce waste from the manufacturing process and are beneficial to increase the capacity utilization of the whole manufacturing process.

Above discussion could be summarized in following hypothesis

- H_1_: Production design has a significant positive relationship with JIT implementation.
- H_2_: Total quality control has a significant positive impact on JIT implementation.
- H_3_: Inventory reduction has a significant positive relationship with JIT implementation.
- H_4_: Supply chain has a significant positive impact on JIT implementation.
- H_5_: Production plan has a significant and positive relationship with JIT implementation.

## Methodology and discussion

### Research design

**Figure 1:**
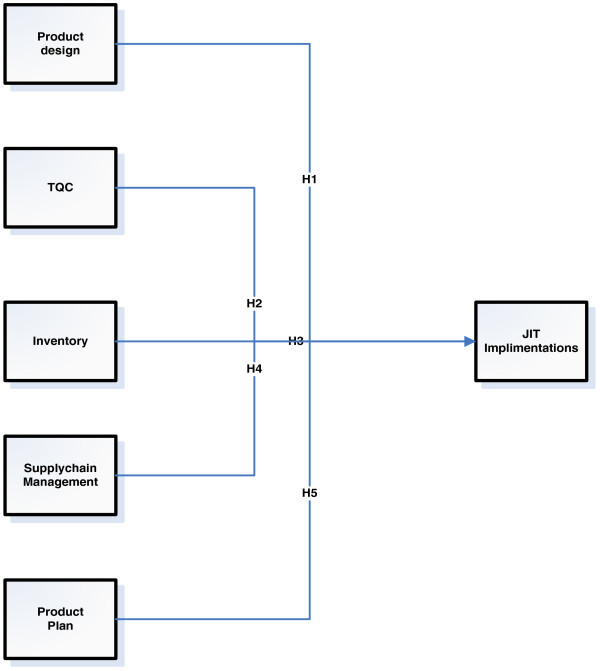
**Research framework.** Source: Self extract.

This study is an ex-post facto, non-experimental design. Ex-post facto is a quantitative research that explores possible causes and effects. Ex-post facto research is used to explore the relationships between independent and dependent variables in all those situations where it is impossible and unethical in order to manipulate the independent variable (Allyn & Bacon 2008). This type of study is very common and useful when using human subjects in real-world situations and the investigator comes in “after the fact” Diem (2002). In this study, an ex-post facto research assists in determining the implementation JIT in cement industry of Pakistan. Figure 1 shows some key factors which may enhance the JIT implementations in an industry.

### Sample selection

The sample of the study consists of operations managers in cement industry of Pakistan. Convenience sampling technique is used for sample selection as convenience sampling involves drawing elements from a group that is easily accessible and it is one of the most commonly used purposive sampling techniques Tashakkori & Teddlie (2003). Data is collected through convenient sample of 400 operations mangers in cement industry of Pakistan. The subjects were 98 per cent male and 2 per cent female. The median age of the sample was 45 years at the time of data collection. Details of the demographic characteristics are given in the Table 1.

**Table 1:** **Demographic characteristics of operations managers**

Respondent’s characteristics	Frequency distribution	Percentages
**Gender**		
Male	392	98%
Female	08	2%
**Designation**		
Production/Operation manager	208	52%
Inventory mangers	124	31%
Plant mangers	68	17%
**Age**		
31–35	160	40%
36–40	136	34%
41–45	80	20%
46–50	16	4%
Above	08	2%
**Provinces**		
Punjab	128	32%
Sindh	96	24%
Baluchistan	48	12%
Khyber Pakhtunkhwa	68	17%
Federal	60	15%

### Data collection instrument

A rigorous study of literature of interest for identifying existing measures to the related constructs was done to finalize questionnaire. The questionnaire was pilot tested on 10 professionals, and then questionnaire is modified to purify the survey that is based on their feedback. In the current study, Likert Scale is being used, as it is the most generally used measure in scale designing like the 3-point and 7-point likert scales commonly enjoying the largest popularities. The 5-point Likert scale for the three major reasons i.e., first, a 5-point Likert Scale is considered to be the most reliable method for measurement as, the questions are over 5, its really difficult for respondents to distinguish the right point. Secondly, 3-points Likert Scale depresses strongest and mildest opinion of people, while a 5-point Likert Scale can express it perfectly. Thirdly, 7-point Likert Scale creates confusion for those people who have weak distinguishing ability. Thus, the study used the 5-point Likert Scale with the responses: 1 as strongly disagrees and 5 as strongly agree.

### Reliability and validity analysis

The reliability of this questionnaire is measured by Cronbach’s coefficient alpha (α). The results show a Cronbach’s alpha score of each dimensional scale i.e., product design has been measure through 3 sub-variables namely; analysis (PD1), concept (PD2) and synthesis (PD3) and have a reliability of 0.72, TQC with 3 sub variables process quality (TQC 1), product quality (TQC 2) and customer satisfaction (TQC 3) at 0.78, Inventory has been measured through 3 sub-variables i.e., Economic order Quantity (INV 1), Continuous ordering (INV 2) and Periodic ordering (INV 3) at 0.71, Supply chain integration sub-variables i.e., distribution network configuration (SCI 1), distribution strategy (SCI 2) and information (SCI 3) at 0.72, product plan has been measured through three indicators resource utilization (PP 1), coordinated activities (PP 2) and Labor productivities (PP 3) at 0.67, JIT Implementation has been measured with six indicators overproduction (JIT 1), waiting (JIT 2), transportation (JIT 3), inappropriate processing (JIT 4), un necessary motion (JIT 5) and defects (JIT 6) at 0.66, while the construct as a whole is at 0.72. This depicts the the sufficient internal consistency of questionnaire, and alpha value is greater than acceptable value of 0.70. Table 2 shows the reliability analysis of each construct.

**Table 2:** **Constructs of reliability analysis**

Construct	No. of Items	Cronbach-alpha	Kaiser-Meyer-Olkin (KMO) test
Production design	3	0.72	0.59
TQC	3	0.78	0.70
Inventory	3	0.71	0.71
Supply chain integration	3	0.79	0.72
Production plan	3	0.67	0.52
JIT implementation	6	0.66	0.57
Over all	21	0.72	0.64

The study adopts factor analysis in order to evaluate the construct validity of the questionnaire, using Kaiser-Meyer-Olkin (KMO) value in the factor analysis, more the KMO value is higher, more correlating factors the variables share, so that, more appropriate is for factor analysis. If the KMO value is more than 0.5 it justifies the use of factor analysis, otherwise it will not be fit for factor analysis. Hence, as Table 2 shows that each variable has the KMO value above 0.5 that shows that each variable is appropriate for factor analysis. It also depicts that the questionnaire has the construct validity which is quite sufficient have, as all the values of factor loadings are above 0.5 reaching to the acceptable range.

### Factor analysis

One of the methods for investigating whether a number of variables of interest are related to the smaller number of unobservable factors linearly or not is Factor analysis. In the unique vocabulary of factor analysis the parameters of these linear functions are termed as loadings. The theoretical variance of every variable and the covariance of every pair of variables could be articulated in terms of the loadings and the variance of the error terms under certain conditions. The communality of a variable is the part of its variance and is explained by the common factors. The specific variance is not be accounted by common factors and is the part of the variance of the variable. An infinite number of sets of loadings are existed that compliant the same theoretical covariance’s and variances.

Factor analysis generally consists of two stages. In first stage, one set of loadings is calculated that shows theoretical variances and covariance’s that fit the observed ones so closely on the basis of certain criterion. These calculated loadings, though, may not be in agreement with the previous expectations, or may not lend themselves to any reasonable interpretation. so, in the second stage, the first loadings are rotated in an effort to reach to another loadings set that fit evenly well the observed variances and covariance’s, but these are more easily interpreted and more are consistent with the prior expectations.

A method broadly used to determine a first set of loadings is called the principal component method. This method found values of the loadings that carry the estimate of the total communality so close to the total of the observed variances. When the variables are not gone to be measured in the similar units it is usual to standardize them prior to the principal component method for subjecting them so that all have mean that is equal to 0 and variance that is equal to 1.

The results of principal component analysis are shown in Table 3. Five factors were extracted by considering that the eigenvalue of the correlation matrix should be more than one, and that the selected factors should explain around 61.66% of the variance. The eigenvalue for a given factor measures the variance in all the variables that is accounted for by that factor (Kim and Mueller 1978).

**Table 3:** **Total variance explained**

Component	Initial eigenvalues			Extraction sums of squared loadings			Rotation sums of squared loadings		
	Total	% of variance	Cumulative %	Total	% of variance	Cumulative %	Total	% of variance	Cumulative %
1	6.817	32.460	32.460	6.817	32.460	32.460	3.095	14.740	14.740
2	1.988	9.468	41.928	1.988	9.468	41.928	3.065	14.595	29.335
3	1.662	7.916	49.844	1.662	7.916	49.844	2.195	10.453	39.787
4	1.297	6.178	56.022	1.297	6.178	56.022	2.135	10.167	49.954
5	1.184	5.639	61.661	1.184	5.639	61.661	2.026	9.648	59.602

Extraction Method: Principal Component Analysis.

The varimax rotation method is related to the detection of factors each of which is relevant to few variables. It discourages the detection of factors that influence all variables. There is substantial subjectivity in determining the number of factors and the interpretation. There are numerous methods in order to obtain the solutions of first and rotated factor, and each solution may provide different interpretation. Table 4 shows rotated component matrix which shows that each item loading on every 5 components of JIT implementation are above 0.50.

**Table 4:** **Rotated component matrix** ^**a**^

	Component				
	Product design	TQC	Product planning	Inventory	Supply chain integration
Analysis	.710				
Concept	.508				
Synthesis	.782				
Process quality		.646			
Product quality		.675			
Customer satisfaction		.754			
EOQ				.707	
Continuous ordering				.784	
Periodic ordering				.737	
Distribution network configuration					.670
Distribution strategy					.433
Information					.541
Resource utilization			.566		
Coordinated work activities			.622		
Labor productivity			.679		
Variation	14.740%	14.595%	10.453%	10.167%	9.648%

^**a**^Rotation Method: Varimax with Kaiser Normalization.

**Figure 2:**
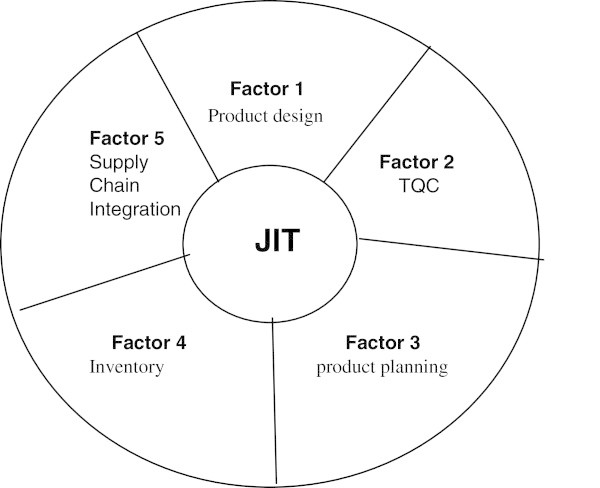
**JIT implementation success model and its practical application.** Source: Self Extract.

The five JIT success factors in order to implement JIT successfully identified in this study formed the JIT success model as depicted in the Figure 2. This table showing the factors determined by the Table 4.

**Figure 3:**
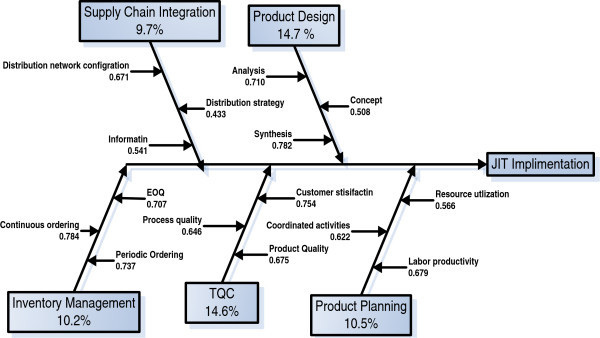
**Factor loaded fish bone.**

Figure 2 shows each factor contribution in JIT implementation process and it can be used as a basis to develop a framework for successful implementation of JIT in different sectors and to improve the effectiveness and efficiency of this process. This may help to formulate effective planning and management of JIT implementation program. Figure 3 shows the fish bone factor loaded diagram for the successful implementation of JIT.

Cause and effect diagrams ually called Fishbone diagram is an analytical decision tool used in the quality management for the analysis of the root cause of the defect. Cause and effect analysis was devised by Professor Kaoru Ishikawa, a pioneer of quality management, in the 1960s. The technique was then published in his 1990 book, “Introduction to Quality Control.” The diagrams that create with Cause and Effect Analysis are known as Ishikawa Diagrams or Fishbone Diagrams (because a completed diagram can look like the skeleton of a fish). Cause and effect analysis was originally developed as a quality control tool; this tool has a variety of uses in the service industry as well to find out the reasons for the lack of quality.

Figure 3 shows that the component of supply chain integration which comprises of three items i.e., distribution network configuration, distribution strategy and information and these items are loaded with the values i.e., 0.671, 0.433 and 0.541. This shows that the component supply chain integration is contributing to the total JIT implementation process with 9.7% of variation caused by this factor. Product design is having total contribution 14.7% variation with three item loadings i-e 0.710, 0.508 and 0.782 in JIT implementation process. Inventory management with the loading values of 0.707, 0.784 and 0.737 that causes overall 10.2% of variation in the process of JIT implementation. TQC causes 14.6% of variation in the JIT implementation. And Product planning causes 10.5% of variation in the data set with three items i.e., resource utilization, coordinated activities and labor productivity having loadings of 0.566, 0.622 and 0.679. Table 5 shows the descriptive statistics and correlation matrix for JIT factors in cement industry.

**Table 5:** **Descriptive statistics and correlation matrix**

JIT factors	Mean	Std. deviation	Product design	Total quality control	Inventory management	Supply chain integration	Product planning	Just in time implementation
Product design	3.65	0.673	1.000					
Total quality control	3.70	0.813	0.450	1.000				
Inventory management	3.87	0.790	0.312	0.610	1.000			
Supply chain integration	3.63	0.745	0.510	0.539	0.437	1.000		
Product planning	3.52	0.671	0.504	0.344	0.356	0.571	1.000	
Just in time implementation	3.62	0.774	0.468	0.456	0.385	0.497	0.362	1.000

Note: correlation is significant at the 0.01 level (2 tailed).

In the above Table 5, it can be seen that the construct i.e., inventory management has the highest mean i.e., 0.387 on the basis of survey responses. In addition, all the constructs have the mean value greater than three showing most of the respondents are agreeing with the statement of the asked questions. The correlation analysis for JIT implementation shows that all the variables i.e., product design, TQC, inventory management, supply chain integration and product planning are showing positive relationships with the JIT implementation as, the relations are significant at p < 0.01.

### Structural equation model (SEM)

Estimation of maximum likelihood is most commonly used technique in SEM Programs. (Hair et al. 2006; Kline 1998). This technique is also used to estimate the value of the unknown parameters in model. It provides a wide range of the outputs like goodness of fit indices, thus giving answers to the questions i.e., whether the model fits or not and to set up the acceptability of the model. Hair et al. (2006) has given few indices that will be used in order judge the fitness of the model.

The model provides an acceptable fit to the data, i.e. the values of CFI, NFI, RMSEA, GFI and AGFI were found according to the cut points in both the measurement models. Model fit was achieved by following an examination of the modification indices. GFI (Goodness of fit index), RMSEA (Root Mean Square Error Approximation) and chi-square statistic are the indices that are commonly used for measures that are called absolute fit measures. These measures determine the degree to which the overall model (structural and measurement models) predicts the observed covariance or correlation matrix. NFI (Normal Fit Index), CFI (confirmatory fit Index) and AGFI (Adjusted Goodness-of-fit Index) are the indices of measures that are called incremental fit measures. These measures compares the proposed model to some baseline model, most often referred to as the null model. The null model should be some realistic model that all other models should be expected to exceed. The standard values of the above mentioned Fitness Indices and values achieved in the research model are shown in Table 6.

**Table 6:** **Model fit summary**

Fitness indices	Standard values	Achieved values
CFI	0.90	.933
NFI	0.90	.894
RMSEA	p < 0.08	.048
GFI	0.80	.877
AGFI	0.80	.794

The value of GFI must exceed 0.80 (Byrne 1998). The value of AGFI must be equal to or greater than 0.80 (Bamber et al. 2000). The Acceptable rang of NFI is greater than or equal to .90 (Gefen et al. 2000). The acceptable value or cut off value for CFI is .90 or higher (Gefen et al. 2000). The acceptable rang for RMSEA changed over the years in fifteen years or so. In early 19^th^ century the range is from 0.05–0.10 and value above 0.010 is considered poor fit MacCallam et al. (1996). RMSEA between .08–0.10 is considered as moderate fit MacCallam et al. (1996) however the most recent cut off points lower limit is considered 0.05 (Gefen et al. 2000) and upper limit of 0.07 (Steiger 2007) is considered general consensus. RMSEA of less than 0.08 suggests a good fit.

The CFI value is 0.973 which is above the 0.9 level used by convention. The Root Mean Square Error of Approximation (RMSEA) for their study was 0.069, which also reveals a very good fit between the model and the data in question. As the above table indicates all the values of Fit indices are well above or equal to the standards. This shows that the model exhibits complete fitness of its variables.

**Figure 4:**
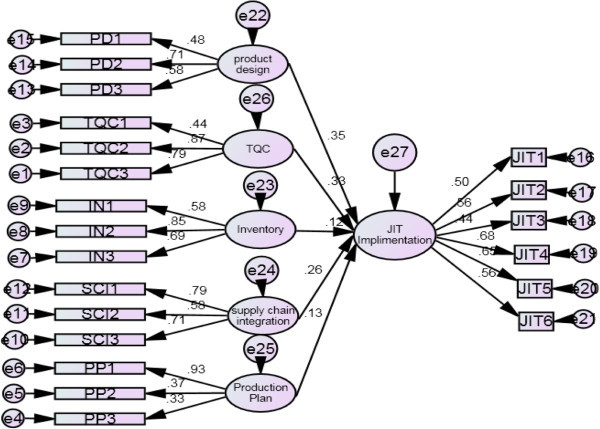
**Structural model of JIT implementation.**

Structural Equation Modeling technique consists of two parts which are performed separately. The first one is measurement model stage, performed to specify how the latent independent variables are measured with respect to Observed dependant variables. The second part of SEM is structural model stage; this stage specifies the interrelationship of latent variables between constructs (Anderson and Gerbing 1988; Hair et al. 2006). This analysis of the two separate models is extremely important (Hair et al. 2006 Kline 1998; Schumacker and Lomax 2004). They are presented as a path diagram because of the complex nature of the models, which highlights the relationship between both the construct and measured variables (Hair et al. 2006). Figure 4 shows the estimations of structural equation model for JIT implementation.

The regression values of the variables support the hypothesis of the study. Regression of product design on JIT is 0.30, which means when JIT increases by 1 standard deviation; product design goes up by 0.30 standard deviations. This supports H1 that product design has significant positive relationship with JIT implementations. TQC has a regression value of .33 which means when JIT increases by 1 standard deviation; TQC goes up by 0.33 standard deviations. This supports H2; TQC has significant positive impact on JIT implementation. Inventory has the value of .12 on the model, which means when JIT increases by 1 standard deviation, Inventory goes up by 0.12 standard deviations supporting H3, and Inventory reduction has significant positive impact on JIT implementation. Supply chain has a value of .26, which means when JIT goes up by 1 standard deviation; Supply chain is increased by 0.26 standard deviations, supporting H4. Production plan has the value of .13 which means when JIT goes up by 1 standard deviation; Production plan goes up by 0.13 standard deviations supporting H5. Similar findings were achieved by Tan (2001), pertaining to the TQC that JIT Strategy significantly influences quality management strategy. Flynn et al. (1995) reveald that Just in time strategies can improve quality by quicker problem exposure, reduced potential for spoilage and improved feedback processes. Germain and Droge (1997) also supports the theory that JIT initiatives leads toward an improved quality because defects and other sources of variance are sought systematically and then eliminated or reduced in a JIT system. Tan (2001), findings also support H1 by showing the results that JIT strategy significantly influences product design and development strategy. Table 7 shows the summary of the hypothesis results.

**Table 7:** **Summary of hypothesis**

Hypothesis	Description	Result
H1	Production design has a significant positive relationship with JIT.	Accepted
H2	TQC has a significant positive impact on JIT.	Accepted
H3	Inventory has a significant positive relationship with JIT.	Accepted
H4	Supply chain has a significant positive impact on JIT.	Accepted
H5	Production plan has a significant and positive relationship with JIT.	Accepted

## Discussion and conclusion

The current study examines those factors which have positive relationship with the implementation of JIT in cement industry of Pakistan. This study focuses the product design, total quality control, inventory management, supply chain integration, production plan and their relationship with the JIT implementation. JIT Implementation is measured through six indicators first **Overproduction** means manufacturing of the products in excessive quantity i-e a huge wastage of time more than demand which results in the wastage of a huge amount of money, space and time as well. Second is important factore is waiting time which reflects the ineffective and inefficient process and the unnaccesary time utilization when in an ongoing process one has to wait for the completion of one process in order to start other. In ideal circumstances, the operations flow must be continuous and smooth. On the basis of some estimates, in manufacturing about ninety nine percent of a product’s time is actually spent waiting, third factor is **Transportation** which means moving a product between different processes of manufacturing that do not add any value. Which in fact is very costly or expensive for any manufacturing plant and it may result in product deterioration or damage, fourth is **Inappropriate processing** which denotes excessively elaborate and luxurious equipment is extravagant if simpler machinery would work as well, fifth is **Unnecessary motion** that shows unusual resources are consumed when workers have to bend, walk or reach distances in order to do their jobs. Workplace ergonomics assessment must be conducted in order to create more proficient environment and the sixth one is **Defect**s which means quarantining and inspecting inventory that takes time and overheads money (Davy et al. 1992).

Product design is measured through a set of indicators which consist of three factors, first ‘*analysis*’ in which general and specific information is collected and on the basis of that information it is attempted to find out about the solution of the problem. Secondly, ‘*concept*’ , in which the basic issue of product designs, is defined. The problem conditions become objectives, and restraints on the situation turn into the parameters in which the new design have to be constructed (Koberg and Bagnell 1991) and third ‘*Synthesis*’ in which manufacturer brainstorm different views and ideas and solutions for the problem of their design (Koberg and Bagnell 1991). An ideal brainstorming session doesn’t contains any judgment or bias but builds on original ideas.

The study found the evidence of positive relationship between JIT implementation and product design with path coefficient of 0.35. It depicts the careful product design could be helpful in implementing JIT in the Pakistani cement industry, which supports hypothesis H1. Manufacturing industry must focus on their product design and need improvements according to the requirements of Just in Time management Concept, which leads to the reduction in the unnecessary movements, inappropriate processing, waiting and certain defects in the production process.

The Factor Total Quality control is also measured through three indicators. First is the ‘*process quality*’ , which focuses overall quality of the process through which product is to be manufactured. Secondly ‘*product quality*’ which indicates the primary characteristics of the product and value addition of the product and third customer satisfaction which relates with the customer perception of the product or fitness for use (Flynn et al. 1995). The study indicates the existence of a direct relationship of TQC with implementation of Just in Time management. So our hypothesis H2 is accepted. TQC can help manufacturing industry in Pakistan to reduced defects which lead to effective implementation of JIT management.

Inventory management is also an important factor to consider for implementation of JIT management philosophy. It was measure through three variables; ‘Economic Order Quantity (*EOQ*)’ which determines optimal order quantity which will reduce the total cost of inventory. EOQ is a basic and essential model and the models that are developed further are based on this very basic model like production quantity model and quantity discount model. **Continuous order** works on the basis of fixed order quantity where trigger is released for fixed quantity replenishment every time the inventory level reaches to the level of predetermined safety and triggers re ordering. **Periodic ordering** works on placing order after a fixed period of time. Study finds that there is a direct relation between JIT and Inventory management and planning (Banerjee and Kim 1995). With the effective management of inventory unnecessary inventory piles and work in progress inventory could be reduced. So manufacturer must focus on inventory management systems to implement JIT and reduce the unnecessary inventory.

Supply chain integration is another important factor that can influence JIT implementation. Supply chain integration is measured through Distribution Network Configuration that is number, facilities of production, location and network suppliers mission, warehouses, distribution centers, cross docks and customers, 2nd Distribution Strategy means operating control questions like if it is centralized, decentralized or shared; delivery scheme for example direct shipment, cross docking, pool point shipping, direct store delivery (DSD), closed loop shipping; transportation mode for example motor carrier, containing truckload, LTL (Less than truckload) parcel; railroad; intermodal transport including TOFC i-e trailer on flatcar and COFE (container on flatcar); airfreight ocean freight; replenishment strategy (for example push, pull, or hybrid); and transportation control (for example owner operated, common carrier, private carrier, contract carrier, or 3rd-party logistics.) and 3rd Information shows an integration of processes by supply chain in order to share information that is more valuable, involving forecasts, demand signals, transportation, inventory and potential collaboration. Study finds a positive relationship with JIT implementation (Cook 1996). Supply chain integration could resolve the inventory problems, can reduce unnecessary motion and defects due to raw materials. Supply chain strategy could be vital to success for implementing JIT.

Production plan is measure through three indicators, first, ‘*resource utilization*’ i.e., how effectively and efficiently resource has been utilized, second, ‘*coordinated activities*’ represents how much work activities are coordinated with each other to minimize the wastage and third, ‘*Labor productivity*’ which leads to the productivity of labor. Product planning has a positive relation with JIT implementation which recommend manufacturers to perform careful product planning in order to reduce the wastage and to improve labor productivity which could lead to effective implementation of Just- in- Time management Philosophy.

In the current situation of Pakistan, it is really a challenge for all production managers to implement the JIT in their production process which leads to zero inventory and make to order production planning. It is also tricky in the prevailing situation and uncertain economic environment of the Pakistan which makes it really impossible in reality and practice. The current study proposed that the integration of product design, quality control, effective inventory management, and production plan and supply chain could overcome these challenge.
